# Milk production and nitrogen excretion of grazed dairy cows in response to plantain (*Plantago lanceolata*) content and lactation season

**DOI:** 10.5713/ab.23.0400

**Published:** 2024-02-23

**Authors:** Thi Truong Nguyen, Soledad Navarrete, David Horne, Daniel Donaghy, Peter Kemp

**Affiliations:** 1 School of Agriculture and Environment, Massey University, Palmerston North 4410, New Zealand; 2 Cargill Vietnam, Ho Chi Minh City 700000, Vietnam; 3 Faculty of Agronomy, University of Concepción, Concepción province, 4030000, Chile

**Keywords:** Milk Production, Plantain Proportion, Urinary Nitrogen, Urine Volume

## Abstract

**Objective:**

The study aimed to quantify milk production and urinary nitrogen (UN) excretion of dairy cows grazing pastures containing varying amounts of plantain (*Plantago lanceolata*) in different seasons, under a typical farm practice.

**Methods:**

Four pasture treatments: perennial ryegrass (*Lolium perenne*) – white clover (*Trifolium repens*) (RGWC), RGWC + low plantain rate, RGWC + medium plantain rate, and RGWC + high plantain rate, were established in four adaptation areas (1 ha each) and 20 experimental plots (800 m^2^ each), and rotationally grazed by dairy cows over 14 grazing events during two lactation years. In each grazing (8 to 9 days), 60 or 80 Jersey-Friesian lactation cows were assigned to their pasture treatments, adapted to their pastures over the first six days, then each group of 15 or 20 cows were randomly allocated for grazing in five treatment plots over a two or three-day measurement period. Milk, urine, and faecal samples were collected from individual cows during the measurement period.

**Results:**

The pasture treatments did not affect milk production, the yield and composition of milk solids, protein, fat, and lactose. However, cows grazing pastures containing between 17% and 28% dietary plantain reduced UN concentration by 15% to 27%, decreased UN excretion by 4% to 9%, and increased urine volume by 22% to 40%, compared to grazing the RGWC pasture. The change in UN concentration, and urine volume were associated with plantain proportion in the diet and were greater during late summer and autumn than during early summer.

**Conclusion:**

Incorporating 17% to 28% dietary plantain with RGWC pastures can reduce the risk of nitrogen losses from pastoral systems, while maintaining the milk production of dairy cows.

## INTRODUCTION

Pastoral dairy production systems in countries, like New Zealand, Australia, Ireland, and some regions in the United States greatly rely on perennial ryegrass (*Lolium perenne*) – white clover (*Trifolium repens*) (RGWC) pastures [1]. These pastures are known for their good persistence and production under grazing conditions that enable high economic performance for dairy farming [2]. However, RGWC pastures often produce low quality and quantity of herbage during hot and dry conditions, leading to feed deficits during summer. In addition, their high nitrogen (N) content can exceed the N requirement of animals, resulting in the risk of N losses from pastoral systems [3]. Recently, governments have developed regulations to reduce the environmental impact of agricultural production [4,5], prompting interest in developing management practices that limit N leaching from dairy systems.

Plantain (*Plantago lanceolata*) is a drought and heat-tolerant forage herb [6] that has increasingly been used in New Zealand grazing pastures for its potential to mitigate environmental impact and improve the productivity of dairy farms [7]. Previous studies have indicated that including plantain in the diet of dairy cows can increase milk yield, milk solids and protein, and reduce urinary nitrogen (UN) excretion [8,9]. Furthermore, the UN concentration, urine volume, and UN excretion of dairy cows can be influenced by the proportion of plantain in their diet [10,11], and can vary across seasons [12,13]. However, these past studies were conducted over short periods, with pastures being cut and fed indoors [10,14], or without supplements while grazing [11,12]. The effect of plantain pastures under these conditions may differ from its actual impact in typical farm practices, where cows are offered both grazing pastures and supplemented feeds.

The advantages of plantain pastures are expected to provide farmers with a cost-effective option to reduce N losses from dairy systems and meet regulatory requirements. Paddock-scale research has demonstrated that dairy cows grazing plantain pastures can halve N leaching losses from dairy systems over an entire lactation season, while maintaining milk solids production compared to RGWC pastures [15]. However, it is important to determine and maintain appropriate plantain proportions to obtain the environmental benefits over extended periods when plantain is incorporated with RGWC in grazing systems [16,17]. Additionally, the composition and quality of plantain can vary significantly across seasons [18], resulting in variable effects on milk production and the UN excretion of dairy cows [12,19]. Therefore, before the widespread adoption of this technology on farms, clear evidence is needed regarding the effect of incorporating plantain in RGWC pasture on milk production and UN excretion of dairy cows under typical farm practices for extended periods. A recent study has indicated that plantain proportion, when incorporated with RGWC under grazed conditions, increases in the first 1.5 years after sowing before declining rapidly in the following year [17]. Furthermore, plantain composition is positively associated with non-structural carbohydrates (NSC), mineral content, and bioactive plant secondary compounds, while negatively correlated with dry matter (DM) and fibre content [17]. Changes in plantain proportion and nutritive value may impact milk production and UN excretion of dairy cows across seasons.

The present study aims to evaluate the effects of grazing pastures incorporated with varying plantain contents on milk yield, milk composition and UN excretion by lactating dairy cows under a typical farm practice over two lactation years. In addition, the study investigates the impact of plantain incorporation on milk production and UN excretion in relation to dietary plantain content and lactation seasons.

## MATERIALS AND METHODS

The study was conducted over two lactation years, from September 2019 to March 2021, at Massey University's No 4 Dairy Farm in Palmerston North, New Zealand (40°23′26.9″ S 175°36′43.5″ E). The research was an on-going measurement of Dairy NZ-led Sustainable Food and Fibre Futures Plantain Potency & Practice programme. The experimental procedures were approved by Massey University Animal Ethics Committee (MUAEC #19/54). The soil test, climate information, pasture establishment and management throughout the experimental period are described by Nguyen et al [17].

### Experimental design and management

The experiment utilised a completely randomised design with repeated measures and a factorial arrangement of treatments. The main factors were pasture treatment, and lactation season, with repeated measurements over several grazing events. Four pasture treatments were randomly established in four adaptation areas (1 ha each) and 20 experimental plots (800 m^2^ each), including: perennial ryegrass (*Lolium perenne*, cv. ONE^50^) and white clover (*Trifolium repens* cv. Tribute) (RGWC), RGWC + low proportion of plantain (*Plantago lanceolata*, cv. Agritonic) (PLL), RGWC + medium proportion of plantain (PLM), and RGWC + high proportion of plantain (PLH). The pastures were rotationally grazed by the dairy cows at 4 to 6 week intervals, resulting in 14 grazing events over the experimental period.

During each grazing period (8 to 9 days), 60 or 80 Jersey-Friesian dairy cows were selected and assigned to their pasture treatments according to milk yield, days in milk, and body weight. The cows were adapted to their pastures over the first six days by strip grazing (approximately 100 or 130 m^2^/cow/d) using temporary electric fences. Then, each group of 15 or 20 cows were randomly assigned to graze in five treatment plots over a two or three-day measurement period (3 or 4 cows per plot). The cows were managed under a typical farm practice, milked twice daily at 07:00 h and 15:00 h, and allocated 14 to 20 kg DM of grazing pasture and 5 to 8 kg DM of concentrated supplements with maize silage, corn gluten pellet, pasture silage, and dried distiller grains.

The pasture area allocated for each day was estimated using a rising plate meter and adjusted every half-day by visually estimating the post-grazing residual. Supplements were fed in a concrete feed pad twice daily after morning milking and before afternoon milking to all the cows in a single group, with over 90% utilisation. Maize silage, corn gluten pellets, pasture silage, and dried distiller grains have respective DM content of 33%, 89%, 38%, and 90%, metabolisable energy (ME) of 10.3, 12.7, 9.0, and 13 MJ/kg DM, and crude protein (CP) of 8.0%, 23%, 15%, and 28% [20]. Fresh water was available *ad libitum* from troughs in each treatment plot and at the feed pad. On average, cows spent approximately 18 hours grazing in the paddock and 6 hours milking and feeding on supplements at the shed. The main characteristics of the animals, feeds, and management during grazing events over the study period are described in Table 1.

### Herbage measurements

Herbage measurements were conducted for individual plots to determine DM mass, DM intake (DMI), and botanical and chemical compositions. For all 14 grazing events over the experimental period, herbage DM mass and DMI were measured by harvesting three quadrat cuts to ground level (0.1 m^2^) a day before grazing for pre-grazing mass and repeating a day after grazing for post-grazing mass. The samples were then oven-dried at 75°C until a constant weight was achieved for determining DM mass. Apparent herbage DMI was estimated for each group of three cows grazing in individual plots using the following equation:


DMI (kg DM/cow/d)=(pre-grazing mass kg DM-post-grazing mass kg DM)/(number of cows×number of grazing days)

Botanical and chemical compositions were measured for ten grazing events that coincided with animal measurements. A hand-plucked sample was taken from each plot at 10:00 h a day before grazing, with 15 to 20 grabs to grazing height. Each sample was thoroughly mixed and divided into two subsamples. The first subsample (approximately 100 g fresh weight) was manually separated into plantain leaves, plantain stems and seed heads (i.e scapes), perennial ryegrass, white clover, weeds, and dead materials. These components were then oven-dried at 75°C until a constant weight was achieved to calculate botanical composition. The second subsample was weighed for fresh weight and then oven-dried at 60°C until a stable weight was achieved to determine DM content. These samples were ground to pass through a 1mm sieve for chemical analysis. The analyses were conducted by a commercial laboratory (Hill laboratory, Hamilton, New Zealand), using near-infrared spectroscopy (NIRS), for CP (N×6.25), crude fat (CF), acid detergent fibre (ADF), neutral detergent fibre (NDF), non-structural carbohydrate (NSC) (NSC = 100 – (CP + ash + CF + NDF), organic matter digestibility (OMD), ME (calculated from OMD), chloride (Cl), potassium (K), sulfur (S), calcium (Ca), and sodium (Na) (nitric acid/hydrogen peroxide digestion). Additionally, the samples were analysed for aucubin and acteoside using high-performance liquid chromatography at Massey University, following the method described in Navarrete et al [21].

### Animal measurements

Animal measurements were conducted to determine milk yield and composition, urine, and faecal characteristics and to estimate urine volume, UN and faecal N (FN) excretions of dairy cows. Milk yield was measured for individual cows in all 14 grazings, using data extracted from a Waikato auto-milking system. Milk samples for milk solids, milk protein and milk urea N (MUN) concentrations were collected in ten grazing events (Table 1). For these grazings, milk samples (approximately 30 ml) were collected from individual cows during morning milk (07:00 h) and afternoon milking (15:30 h) on day one and day eight of the grazing periods. These samples were analysed for milk solids (vacuum oven, AOAC 990.19, 990.20), the content of protein (protein = 6.25 × N), fat, and lactose, and MUN (Urease Kinetic UV assay), in the Nutrition Laboratory, Massey University, Palmerston North, New Zealand.

The samples of urine and dung were from individual cows in six grazings during the late lactation period (Table 1). Urine and faecal samples were collected after morning milking on days 7 and 8 of the grazing periods. Urine samples (approximately 80 mL each) were taken by vulva stimulation and subsampled into two smaller samples of about 30 mL each. The first subsamples were acidified with sulfuric acid (6.0 normal) to reduce the pH between 3.0 to 4.0 and analysed for N concentration using the Dumas method. The second subsamples were analysed for creatinine content (Jaffe method), and Na, K, and Cl (ion-selective electrode method). Faecal samples were taken by rectal stimulation and recorded for fresh weight. These samples were then freeze-dried until a constant weight was achieved to estimate the DM content. The dried samples were ground through a 1 mm sieve and then analysed for N concentration (Dumas method) and non-protein N (colourimetric method). The analysis of urine and faecal samples was conducted by the Nutrition Laboratory at Massey University. Urine volume, UN excretion, and FN excretion were estimated using the following equations:

**Table:** 

Urine volume (kg/d)=21.9×body weight (kg)×(1/urinary creatinine (mg/kg))	(Paceco et al [ 22])
UN excretion (g/d)=urinary volume (kg)×UN (g/kg))	(Paceco et al [ 22])
FN excretion (g/d)=FN (g/kg)×DMI (kg/d)×(1-digestibilit (%))	(Ahvenjärvi et al [ 23])

### Statistical analysis

Statistical analysis was performed using the PROC mixed procedure in SAS [24] to analyse differences between pasture treatments in milk, urine, and faecal variables. The model used for analysis was as follows:


$${\text{Y}}_{\text{ijkl}}=\mu +{\text{T}}_{\text{i}}+{\text{S}}_{\text{i}}+{\text{Y}}_{\text{k}}+{\left({\text{T}}_{\text{i}}\times \text{S}\right)}_{\text{ij}}+{\left(\text{T}\times \text{Y}\right)}_{\text{ik}}+{\left(\text{S}\times \text{Y}\right)}_{\text{ik}}+{\left(\text{T}\times \text{S}\times \text{Y}\right)}_{\text{ijk}}+{\text{e}}_{\text{ijk}},$$

where, Y_ijk_ = dependent variable; μ = overall mean; T_i_ = pasture treatment i; S_j_ = season j (spring = September to November, early summer = December to January, late summer = February, Autumn = March); Y_k_ = year k; (T × S)_ij_ = the interaction between pasture treatment i and season j; (T × Y)_ik_ = the interaction between pasture treatment i and year k; (S × Y)_jk_ = the interaction between season j and year k; (T × S × Y)_ijk_ = three-way interaction between pasture treatment i, season j and year k; e_ijk_ = residual error.

Furthermore, the relationships between dietary plantain (% plantain leaves) and the relative change (RC) in UN concentration and urine volume in comparison to RGWC were examined. The analysis was conducted using the PROC reg procedure in SAS [24]. Relative change in a variable was estimated using the equation: RC = 1 – Y_PLi_/Y_RGWC_, where Y_PLi_ and Y_RGWC_ refer to the mean value of treatment i and RGWC, respectively. Statistical significance and tendency were declared at p<0.05 and 0.05≤p<0.10, respectively.

## RESULTS

### Herbage characteristics

Pasture treatment had a significant effect (p<0.05) on all botanical and chemical composition variables, except for white clover, weeds, and CP content (Table 2). Plantain content was on average 5% in RGWC, 32% in PLL, 44% in PLM and 48% in PLH. On average, plantain comprised 76% of leaves and 24% of reproductive stem and seed head. Perennial ryegrass contributed 62% in RGWC, 39% in PLL, 29% in PLM, and 26% in PLH (p<0.05). White clover content was between 14% and 16%, while weeds accounted for less than 1.3% for all treatments.

For chemical composition, pastures containing between 31% and 48% plantain had a higher content of NSC by 10% to 24%, OMD by 3% to 4%, Ca by 41% to 73%, Na by 43% to 66%, and Cl by 22% to 33%, but contained a lower DM content by 16% to 24%, along with CF by 4% to 7%, ADF by 3% to 7%, and NDF by 6% to 13%, compared to RGWC. In addition, the plantain pastures contained between 3.4 to 5.9 g/kg DM aucubin and 5.2 to 10.2 g/kg DM acteoside. There was no significant difference in CP and ME between treatments.

### Milk yield and milk composition

Pasture treatment did not affect herbage DMI, milk yield, the composition and yield of milk solids, milk protein, milk fat, or milk lactose (p>0.05); however, the plantain treatments significantly decreased the content of MUN in comparison to RGWC (p<0.05) (Table 3). On average, cows consumed 3.7, 5.7, and 6.4 kg DM plantain per day in PLL, PLM, and PLH, accounting for 17%, 25%, and 28% of the apparent diet, respectively. The average MUN of cows grazing plantain pastures was lower than that in RGWC by 10% to 17% (p<0.05).

### Urine production and nitrogen excretion

Cows grazing plantain pastures had a lower UN concentration by 15% to 27%, and a lower urinary creatinine concentration by 17% to 27%, compared with those grazing RGWC (p<0.05) (Table 4). The lower urinary creatinine was reflected in the higher urine volume of 22% to 40% from cows grazing plantain pastures (p<0.05). Furthermore, grazing plantain pastures caused an increase of 4% to 9% in faecal N concentration (p<0.05) and a decrease of 1% to 6% in faecal DM content (p<0.05) of cows. Urinary Na, K, and Cl excretion of cows grazing plantain pastures were higher than from those grazing RGWC (p<0.05). All plantain treatments significantly affected UN concentration, urinary creatinine, urine volume, Na, K, and Cl excretion in the urine, and faecal N concentration. However, only the medium (PLM) and high (PLH) rates of plantain reduced faecal DM content (p<0.05).

In cows grazing plantain pastures, N partitioned to urine decreased (p<0.05) and N partitioned to dung increased (p<0.05), but N partitioned to milk was unaffected (p>0.05) (Table 4). Specifically, cows grazing plantain pastures had a 4% to 9% lower UN excretion and a 3% to 7% higher FN excretion than those grazing RGWC (p<0.05). Among treatments, the medium (PLM) or high rate (PLH) of plantain decreased UN excretion (p<0.05), but only the high rate of plantain (PLH) significantly increased faecal N excretion.

### Urine volume and urine N concentration in different lactation seasons

**Figure 1:**
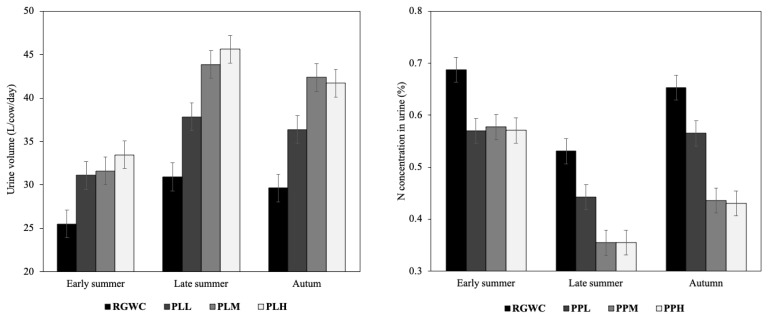
Urine volume and nitrogen (N) concentration in the urine of cows grazing perennial ryegrass-white clover (RGWC), RGWC + low plantain rate (PLL), RGWC + medium plantain rate (PLM) and RGWC + high plantain rate (PLH) in lactation periods.

The results in Table 4 and Figure 1 show that the seasonal effect of plantain pastures on urine production and N concentration in urine was stronger during late summer and autumn than during early summer (p<0.05). In early summer, grazing PLL, PLM, and PLH pastures increased urine volume by 24%, 26%, and 35%, respectively and decreased UN concentration by 17%, 16%, and 17%, respectively, when compared to RGWC. Specifically, the increase in urine volume by grazing PLL, PLM and PLH pastures was 22%, 42%, and 47% in late summer, respectively, and 23%, 42% and 40% in autumn. Meanwhile, the reduction in UN concentration was 17%, 33%, 33% in late summer, and 13%, 33%, and 34% in autumn for cows with PLL, PLM and PLH, respectively, compared to the UN concentration of cows grazing RGWC.

### Relationship between plantain content and urine production

**Figure 2:**
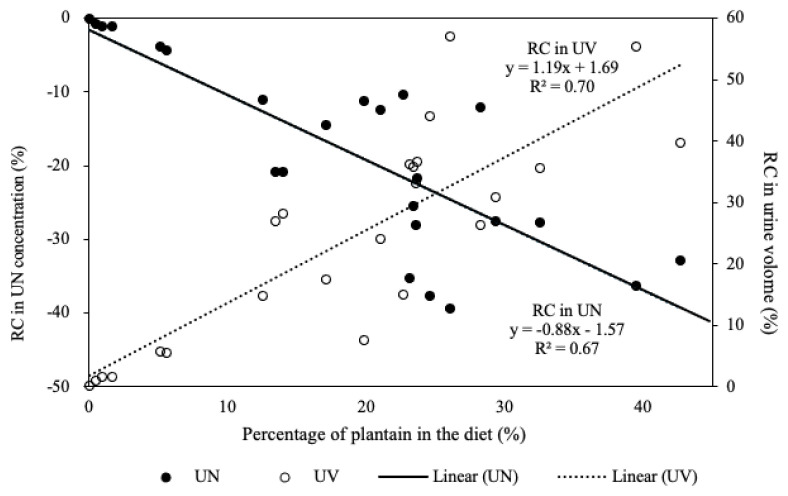
The relationship between the content of dietary plantain leaves and relative change (RC) in urinary nitrogen concentration (UN) and RC in urine volume (UV) over the study period.

The correlation analysis showed that plantain (leaves) content was negatively associated with UN concentration (p<0.05) and positively correlated with urine volume (p<0.05). Additional regression analyses provided linear regression models to estimate the change in urine volume (RC in urine volume (%) = 1.19 × plantain in the diet (%) + 1.69) and in N concentration in urine (RC in UN concentration (%) = −0.88 × plantain in the diet (%) – 1.57) (Figure 2). In these models, the percentage of plantain in the diet explained 70% of the change in urine volume, and 67% of the change in UN concentration.

## DISCUSSION

Previous studies have identified that including plantain in the diets of dairy cows has potential to reduce the concentration of N in their urine, decrease UN excretion, increase urine volume, and improve milk yield in short-term research [8,9]. The lower UN concentration and decreased excretion from cows grazing plantain-based pastures expose a beneficial environmental trait that helps reduce nitrate leaching, thereby contributing to the mitigation of ground and surface water contamination [25]. However, to give farmers greater confidence in integrating plantain with traditional pastures, it is essential to study the effect of plantain pastures on milk production, and changes in urine N loading dynamics (urine volume and UN excretion) on lactating dairy cows under farm conditions over an extended period. In the present study, more pronounced increases in urine volume and decreases in UN concentration were observed in late summer and autumn.

Including plantain in pastures can increase or maintain milk yield and milk composition, depending on the difference in DMI and herbage quality of the pastures and the research conditions [8,26]. In the present study, cows grazing plantain pastures maintained milk yield and composition over two lactation years. These results aligned with those reported by Navarrete et al [19] and Nkomboni et al [11] which showed, under grazing conditions, including plantain in the diet maintained milk production. The plantain content in grazing pastures is usually low and likely insufficient to boost the DMI of cows to produce an more milk, especially considering the variations in pasture quality and environmental factors during different grazing events and measurements [16]. In the present study, the lack of differences in milk production between cows grazing plantain-RGWC pasture and RGWC pastures can be attributed to the comparable levels of both DMI and ME intake in the two groups.

The long-term effects of plantain inclusion on increasing urine volume and decreasing UN concentration and excretion by lactating cows in the present study were similar to previous short-term research [10,27]. Over the two lactation years measured, cows grazing plantain-RGWC produced more urine than cows grazing RGWC, when the percentage of plantain comprised between 17% and 28% of the diet, supporting the dilution effect of plantain reducing the UN concentration. The research data confirmed that the increase in urine volume and the decrease in UN concentration were associated with the proportion of dietary plantain [8,10]. With the R^2^ of 0.70 and 0.67, the developed linear regression models in the present study (in Figure 2) provide feasible estimations for the relative change in urine volume and UN concentration when the plantain proportion in the diet is identified. Especially, each percent of dietary plantain can cause about 0.88% reduction in UN% and 1.16% increase in urine volume. These models provide estimations for the plantain effect under grazing conditions with comparable accuracies to the models developed by meta-analyses of Nguyen et al [8] and Nkomboni et al [11].

The effect of plantain increasing urine production is attributed to the higher water content in plantain herbage compared to RGWC pastures [10,27,28]. In the present study, the water intake estimated using the DM content and DMI of cows grazing in the plantain pastures was 11L-23L/cow/day higher when compared to cows grazing RGWC pastures. The additional water intake from the plantain herbage at least partly explained the increase in urine volume of 7 to11 L/cow/d, when plantain was part of cows diet [27,28]. The higher mineral content in plantain has been proposed as a factor that might require more urine volume to be produced by cows to excrete the surplus minerals [27,29].

In the present study, the average effective dietary plantain in an extended period to increase urine volume and decrease UN excretion were 17% and 25%, respectively. These plantain proportions are comparable to the 18% and 25% of plantain in the diet reported by Nguyen et al [28]. The effect of plantain on reducing UN excretion has been also suggested to be influenced by the presence of plant secondary compounds in plantain. The most well-known bioactive compounds in plantain, aucubin and acteoside, have been shown to inhibit ammonia production during rumen fermentation, suggesting to a lower urea production in the cow rumen fluid [21]. The higher concentration of NSC and non-degraded N in plantain can also reduce UN excretion and N partitioning to urine, while increasing N partitioning to milk and faeces [26,30].

The decreased UN concentration and UN excretion from cows grazing plantain pastures can lead to decreased N leaching from dairy farms. Urine N more readily leads to nitrate leaching than faecal N [31]; therefore, the reduction in UN excretion leads to a decrease in the amount of N vulnerable to nitrate leaching [25,32]. Meanwhile, the increase in FN excretion means that more N could be converted into organic N in soils and used by pastures. The greater urine volume and the lower UN concentration mean that cows can deposit urine N onto a larger soil area with a lower N loading rate. As a result, pastures can uptake more urine N from pastoral soils, leading to less N to be leached into the environment [3,33]. Using the APSIM model, Ledgard et al [34] found that at a similar amount of urine N excretion, a reduction in nitrate leaching by 50% from urine patches and by 22% from grazed paddocks resulted when the UN concentration was diluted (by increasing urine volume) to a 40% lower UN concentration. The effect of plantain on N losses from pastoral systems is associated with different N processes between pastures, animals and soils [25]. The present study only quantified the effect of plantain on N excretion by grazing cows. Therefore, direct measurements at the paddock scale are required to evaluate the impact of plantain pastures on nitrate leaching from pastoral systems and its association with different N components, such as N uptake by plants, nitrous oxide emission, nitrification and denitrification processes in soils.

As previous reports [12,19], the effect of plantain on urine volume, UN concentration and UN excretion is stronger during late summer and autumn than during early summer. The difference in the effect of plantain across seasons was likely due to the greater plantain proportion and greater difference in pasture quality between plantain and RGWC in periods of restricted soil moisture. As a drought and heat-tolerant herb specie, plantain often grow well and produces more DM yield in drier and hotter seasons [17]. However, the high proportion of plantain reproductive stem and seed heads in early summer may reduce the intake of plantain because cows prefer to select palatable ryegrass and plantain leaves rather than the lower-quality plantain stem and seed heads [35]. The differences in DM content, bioactive compounds, and carbohydrate profiles between plantain and RGWC have been reported to be greater in drier seasons [17]. The greater effect of plantain pastures on reducing UN excretion and increasing urine volume in late lactation is likely to lead to reductions in annual N leaching from pastoral systems. The deposition of N in urine patches in late summer and early autumn is the major source of N losses [36]. Therefore, maintaining a high plantain content in the diet of dairy cows during summer and autumn would be important to enhance the benefits of plantain incorporation.

## CONCLUSION

Incorporating plantain with RGWC can increase urine volume, reduce N concentration in urine, and maintain milk yield and milk compositions under farm conditions. The present study found that maintaining an average of 17% plantain in the diet for an extended period can reduce UN concentration and increase the urine volume of grazing dairy cows. On average, each percent of dietary plantain results in about 0.88% decrease in UN% and 1.16% increase in urine volume. The changes in UN concentration and urine volume were linear with the percentage of plantain in the diet of cows. In addition, the effect of plantain pastures on UN concentration and urine volume in late summer and autumn was stronger than in early summer. These findings suggest that maintaining approximately 20% dietary plantain could be a feasible strategy to reduce the risk of nitrate leaching from pastoral systems.

## Figures and Tables

**Table 1:** The summary of animals, feeds, and measurements during grazing events over the experimental period

Grazing period	No. cows	Milk yield (L/d) (Mean±SD)	Plantain leaves (% in pastures) (PLL:PLM:PLH)^1)^	Herbage intake (kg DM/d)(mean±SD)	Supplement (kg DM/d)	Measurement^2)^
2019/2020						
12–20/09	80	35.4±5.4	12:21:35	17.4±2.2	7.5	Y, C
26–24/10	80	32.8±5.4	11:19:28	18.8±4.4	7.5	Y
13–21/11	80	31.6±4.7	15:22:31	14.0±2.3	7.5	Y
14–12/12	80	30.7±4.2	18:29:30	13.8±2.2	6.0	Y, C, A
9–17/1	80	26.5±3.3	28:31:39	17.8±2.6	6.0	Y
5–12/2	60	23.5±3.4	28:37:41	18.3±4.8	6.0	Y, C, A
2–19/3	60	17.4±2.9	40:57:61	15.8±3.9	7.5	Y, C, A
2020/2021						
27/8–5/9	60	38.8±5.5	42:63:61	14.1±3.0	8.0	Y, C
7–15/10	80	33.2±4.7	31:47:46	16.7±2.4	6.5	Y, C
11–19/11	80	29.3±4.7	23:28:30	14.3±1.9	8.0	Y, C
2–10/12	60	27.5±3.6	17:29:30	18.5±2.5	8.0	Y, C, A
6–14/1	80	24.6±3.4	11:23:22	13.9±2.0	5.0	Y
3–10/2	60	23.1±3.3	18:31:33	17.2±2.9	5.0	Y, C, A
24–31/3	60	18.2±2.0	21:30:29	16.2±2.7	5.0	Y, C, A

SD, standard deviation; DM, dry matter.

1) PLL, perennial ryegrass and white clover (RGWC) + low plantain rate; PLM, RGWC + medium plantain rate; PLH, RGWC + high plantain rate.

2) Y, milk yield; C, milk composition; A, urine and faecal nitrogen.

**Table 2:** Botanical and chemical compositions of treatment

Items	Treatment^1)^				SEM	p-value^2)^			
	
	RGWC	PLL	PLM	PLH		T	T×S	T×Y	T×S×Y
Pre-grazing (1,000 kg DM/ha)	3.56	3.51	3.35	3.52	0.09	0.348	0.989	0.925	0.944
Post-grazing (1,000 kg DM/ha)	2.03^a^	1.93^ab^	1.77^b^	1.86^b^	0.07	0.043	0.380	0.073	0.350
Plantain leaves (g/kg DM)	33^a^	238^b^	345^c^	376^c^	12.9	<0.001	0.145	0.243	<0.001
Plantain reproductive stem (g/kg DM)	12^a^	79^b^	95^b^	102^b^	9.4	<0.001	0.008	0.347	0.761
Total plantain (g/kg DM)	45^a^	317^b^	440^c^	478^c^	16.9	<0.001	0.77	0.023	<0.001
Perennial ryegrass (g/kg DM)	620^a^	388^b^	289^c^	260^c^	26.7	<0.001	0.213	0.039	<0.001
White clover (g/kg DM)	164	145	157	144	17.1	0.813	0.776	0.009	0.107
Weeds (g/kg DM)	11	13	8	11	3.1	0.733	0.598	0.890	0.195
Dead materials (g/kg DM)	160^a^	137^b^	106^c^	106^c^	4.9	<0.001	0.117	0.513	0.793
DM (g/kg FW)	270^a^	228^b^	208^c^	204^c^	3.3	<0.001	<0.001	0.590	0.022
CP (g/kg DM)	183	186	189	189	2.8	0.361	0.920	0.220	0.827
CF (g/kg DM)	28^a^	27^b^	27^b^	26^b^	0.4	<0.001	0.258	0.483	0.706
ADF (g/kg DM)	269^a^	261^b^	254^c^	251^c^	2.7	<0.001	0.760	0.545	0.521
NDF (g/kg DM)	469^a^	443^b^	408^c^	407^c^	5.9	<0.001	0.983	0.384	0.639
NSC (g/kg DM)	210^a^	232^b^	259^c^	260^c^	4.6	<0.001	0.897	0.502	0.601
OMD (g/kg DM)	704^a^	722^b^	731^b^	730^b^	6.0	0.006	0.498	0.760	0.991
ME (MJ/kg DM)	10.1	10.2	10.4	10.4	0.07	0.075	0.384	0.814	0.696
Ca (g/kg DM)	8.3^a^	11.7^b^	14.3^c^	14.4^c^	0.47	<0.001	0.270	0.264	0.145
K (g/kg DM)	23.5	23.9	24.1	24.9	0.61	0.418	0.515	0.013	0.842
Na (g/kg DM)	3.5^a^	5.0^b^	5.8^b^	5.7^b^	0.29	<0.001	0.033	0.102	0.018
Cl (g/kg DM)	12.3^a^	15.0^b^	16.3^b^	16.3^b^	0.62	<0.001	0.352	0.853	0.335
Aucubin (g/kg DM)	0.62^a^	3.36^b^	5.20^c^	5.93^c^	0.25	<0.001	0.002	<0.001	<0.001
Acteoside (g/kg DM)	0.6^a^	5.2^b^	9.1^c^	10.2^c^	0.6	<0.001	0.004	<0.001	<0.001

SEM, standard error of the means; DM, dry matter; FW, fresh weight; CP, crude protein; CF, crude fat; ADF, acid detergent fibre; NDF, neutral detergent fibre; NSC, non-structural carbohydrate; OMD, organic matter digestibility; ME, metabolisable energy; DMI, DM intake.

1) RGWC, perennial ryegrass – white clover; PLL, RGWC+low plantain rate; PLM, RGWC + medium plantain rate; PLH, RGWC + high plantain rate (PLH) over the experimental period.

2) T, treatment; S, season; Y, lactation year.

a–c Means within a row with different superscripts differ at p<0.05.

**Table 3:** Milk yield, milk solids, protein, fat, lactose, and milk urea nitrogen of dairy cows grazing treatment

Items	Treatment^1)^				SEM	p-value^2)^			
	
	RGWC	PLL	PLM	PLH		T	T×S	T×Y	T×S×Y
Herbage intake (kg DM/d)	15.5	16.0	16.4	17.1	0.49	0.129	0.704	0.780	0.930
Total apparent intake (kg DM/d)	21.3	21.7	22.2	22.8	0.50	0.135	0.760	0.785	0.945
Dietary herbage content (%)	72.8	73.1	73.7	74.5	0.60	0.176	0.609	0.909	0.940
Herbage N intake (g N/d)	456	456	478	497	15.2	0.164	0.325	0.560	0.676
Plantain intake (kg DM/d)	0.53^a^	3.74^b^	5.71^c^	6.40^c^	0.24	<0.001	0.108	0.448	0.004
Plantain in the diet (%)	2.4	17.0	25.3	27.8	0.96	<0.001	0.103	0.356	0.001
Milk yield (L/d)	25.5	25.6	25.7	25.9	0.25	0.698	0.396	0.882	0.337
Milk solid (%)	13.7	13.8	13.7	13.8	0.09	0.623	0.058	0.141	0.009
Milk solids (kg/d)	3.33	3.35	3.36	3.40	0.04	0.665	0.221	0.245	0.076
Protein (%)	3.43	3.47	3.41	3.46	0.02	0.081	0.387	0.575	0.196
Protein (kg/d)	0.84	0.85	0.84	0.86	0.01	0.310	0.045	0.950	0.479
Fat (%)	4.20	4.17	4.17	4.25	0.09	0.930	0.555	-	-
Fat (kg/d)	1.03	1.01	1.02	1.04	0.02	0.881	0.053	-	-
Lactose (%)	4.98	4.98	4.96	4.97	0.02	0.788	0.779	-	-
Lactose (kg/d)	1.34	1.35	1.35	1.36	0.02	0.914	0.440	-	-
MUN (mg/dL)	10.2^a^	9.2^b^	8.5^c^	8.7^c^	0.10	<0.001	0.001	0.001	0.001

SEM, standard error of the means; DM, dry matter; MUN, milk urea nitrogen.

1) RGWC, perennial ryegrass – white clover (RGWC); PLL, RGWC + low plantain rate; PLM, RGWC + medium plantain rate; PLH, RGWC + high plantain rate (PLH) over the experimental period.

2) T, treatment; S, season; Y, year.

a–c Means within a row with different superscripts differ at p<0.05.

**Table 4:** Urine volume, urine and faecal composition, and nitrogen (N) excretion of dairy cows grazing treatment

Items	Treatment^1)^				SEM	p-value^2)^			
	
	RGWC	PLL	PLM	PLH		T	T×S	T×Y	T×S×Y
Urine									
Urine volume (L/d)	28.7^a^	35.1^b^	39.3^c^	40.3^c^	1.4	<0.001	0.073	0.011	0.513
N (%)	0.62^a^	0.53^b^	0.46^c^	0.45^c^	0.02	<0.001	0.024	0.680	0.075
Urea (mmol/L)	220^a^	193^b^	170^c^	178^c^	4.8	<0.001	<0.001	0.246	0.238
Creatinine (mmol/L)	4.01^a^	3.31^b^	3.00^c^	2.93^c^	0.09	<0.001	0.207	0.757	0.240
Na (g/d)	54.5^a^	70.4^b^	83.1^b^	78.3^ab^	3.0	<0.001	0.002	0.483	0.004
K (g/d)	203^a^	234^b^	255^c^	270^d^	5.2	<0.001	0.021	0.010	<0.001
Cl (g/d)	133^a^	201^b^	212^bc^	225^c^	5.8	<0.001	<0.001	0.005	<0.001
Faeces									
DM content (%)	12.0^a^	12.1^ab^	12.5^bc^	12.7^c^	0.22	0.053	0.289	0.639	0.088
N (%)	2.43^a^	2.53^b^	2.55^b^	2.64^c^	0.02	<0.001	0.872	0.561	0.714
FNP (%)	0.19^a^	0.21^b^	0.20^b^	0.20^b^	0.003	0.001	0.196	0.300	0.032
N excretion									
Urine N (g/d)	180^a^	174^ab^	164^b^	167^b^	3.6	0.004	0.012	0.029	0.373
Milk N (g/d)	116	119	121	119	1.7	0.094	0.009	0.862	0.130
Faecal N (g/d)^3)^	121^a^	124^a^	124^a^	130^b^	1.4	<0.001	0.317	0.842	<0.001

SEM, standard error of the means; DM, dry matter; FNP, faecal non-protein nitrogen.

1) RGWC, perennial ryegrass – white clover (RGWC); PLL, RGWC + low plantain rate; PLM, RGWC + medium plantain rate; PLH, RGWC + high plantain rate (PLH) over the experimental period.

2) T = treatment, S = season, Y = year.

3) Faecal N excretion was estimated using the total DMI, including herbage and supplement intake, assuming cows consumed 90% of offered supplements.

a–d Means within a row with different superscripts differ at p<0.05.
